# Spontaneous closure of a metachronous brochopleural fistula after omentoplasty for a preceding fistula: Case report

**DOI:** 10.1016/j.amsu.2022.104645

**Published:** 2022-09-13

**Authors:** Shuhei Iizuka, Asuka Uebayashi, Toru Nakamura, Kazuhito Funai

**Affiliations:** a Department of General Thoracic Surgery, Seirei Hamamatsu General Hospital, 2-12-12 Sumiyoshi, Naka-ku, Hamamatsu City, Shizuoka, 430-8558, Japan; b First Department of Surgery, Hamamatsu University School of Medicine, 1-20-1 Handayama, Higashi-ku, Hamamatsu City, Shizuoka, 431-3192, Japan

**Keywords:** Metachronous, Bronchopleural fistula, Omentoplasty, Case report

## Abstract

**Introduction:**

A bronchopleural fistula (BPF) after an anatomical lung resection commonly arises singly. We report a case of a metachronous BPF, which developed after omentoplasty of a preceding fistula and subsequently closed without any intervention.

**Case presentation:**

A 77-year-old patient underwent omentoplasty for a brochopleural fistula (BPF) following a right lower lobectomy. A sudden massive air leak developed from the novel BPF approximately 1 cm proximal to the preceding fistula 3 days later. The air leak resolved spontaneously without any intervention one week later. The corresponding fistula was found to be completely closed. Computed tomography showed the omental flap covered both fistulae.

**Conclusion:**

The present case suggested that a metachronous BPF could develop and a harvested omental flap might migrate even after being anchored.

## Highlights

• A metachronous bronchopleural fistula (BPF) developed 3 days after an omentoplasty for a preceding fistula following a right lower lobectomy.
• The BPF was closed spontaneously without any subsequent intervention and a computed tomography revealed that the omental flap covered both fistulae.
• A metachronous BPF could develop and a harvested omental flap might migrate even after being anchored.

## 1 Introduction

A bronchopleural fistula (BPF) is one of the most serious complications after an anatomical lung resection [1]. It commonly arises singly and there have been no reports of metachronous multiple cases to date. Since the BPF is often accompanied by empyema, the treatment strategy could vary widely, and omentoplasty as a one-stage surgery is a feasible alternative to an invasive open window thoracostomy [[1], [2], [3], [4], [5]]. We herein report a case of a metachronous BPF, which developed after omentoplasty of a preceding fistula that subsequently closed without any intervention. This case report has been reported in line with the SCARE Criteria [6].

## 2 Case Presentation

A 77-year-old patient developed a BPF and sepsis due to empyema 11 days after a right lower lobectomy for primary lung cancer. The patient had a history of lumber spinal stenosis and osteoarthrosis but not any history of medications or smoking. The family history was noncontributory.

The patient successfully received suctioning pleural drainage for a postoperative chylothorax and low-fat diet and octreotide from 7 days prior. Omentoplasty was planned to simultaneously resolve the BPF and empyema as a one-stage surgery.

The omeontoplasty was performed under general anesthesia. A pedicled omental flap was harvested laparoscopically and lifted into the right thoracic cavity through the diaphragm in the supine position. Then, a thoracoscopic procedure was performed in the left lateral decubitus position. An intrathoracic view revealed a fistula of the membranous portion of the bronchial stump with an acute phase of empyema. The omental flap was fixed to the bronchial stump by interrupted sutures with two non-absorbable monofilament threads. The operative time was 265 minutes with 105 ml of blood loss. The patient required mechanical ventilation after the omentoplasty because of acute respiratory failure due to fluid aspiration from the BPF.

**Fig. 1:**
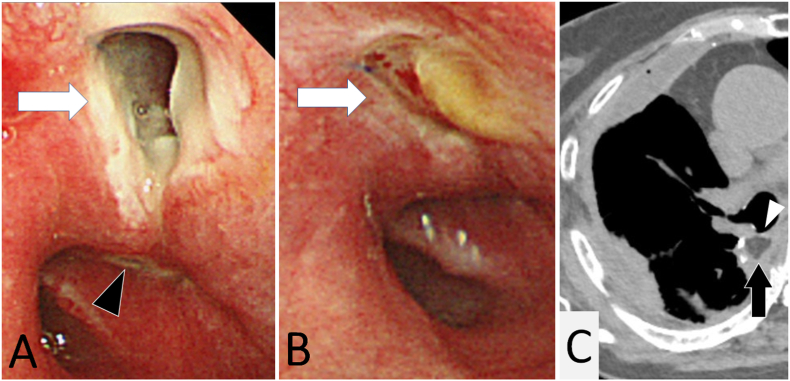
Development of a novel fistula and its healing process. A: Video bronchoscopy 3 days after the omentoplasty revealed a novel fistula (arrow) 1 cm proximal to the closed preceding fistula (arrowhead). B: The novel fistula (arrow) was found to have closed spontaneously a week later. C: A chest CT revealed that the omental flap (black arrow) completely covered both fistulae (white arrowheads).

A sudden massive air leak developed 3 days later, and a novel BPF was found approximately 1 cm proximal to the closed fistula (Fig. 1A). The patient refused any further surgical intervention and was managed with only chest tube drainage. Despite successful weaning from mechanical ventilation, the patient required a surgical tracheotomy to facilitate efficient sputum suctioning.

The air leakage resolved spontaneously a week after the omentoplasty. The corresponding fistula, as well as the preceding one, was found to have completely closed (Fig. 1B). Computed tomography showed that the omental flap had obliterated the entire empyema cavity covering both fistulae (Fig. 1C). The patient was discharged without any sequelae 48 days after the omentoplasty for ambulatory follow-up at six month intervals and is currently disease free at 19 months. All treatments were performed by board certificated thoracic surgeons at one district general hospital.

## 3 Discussion

The incidence of BPF after anatomical lung resections and its mortality rate are 1–4% and 18–50%, respectively [1,[7], [8], [9]]. The risk factors for a BPF include a right lower lobectomy, pneumonectomy, positive pressure ventilation, bronchial ischemia, and malnutrition [1,3,7,10]. Surgery remains the mainstay of the treatment and omentoplasty is one of the most promising surgical interventions because it could resolve the BPF and empyema simultaneously [[1], [2], [3], [4], [5]]. It is also beneficial for improving the patients’ quality of life compared to the open window thoracostomy of which daily gauze dressings are required for a certain period of time [2]. The omentum has not only a volume-filling effect, but has an excellent immunological and angiogenic ability that promotes wound healing and suppresses infections [2,[11], [12], [13]].

**Fig. 2:**
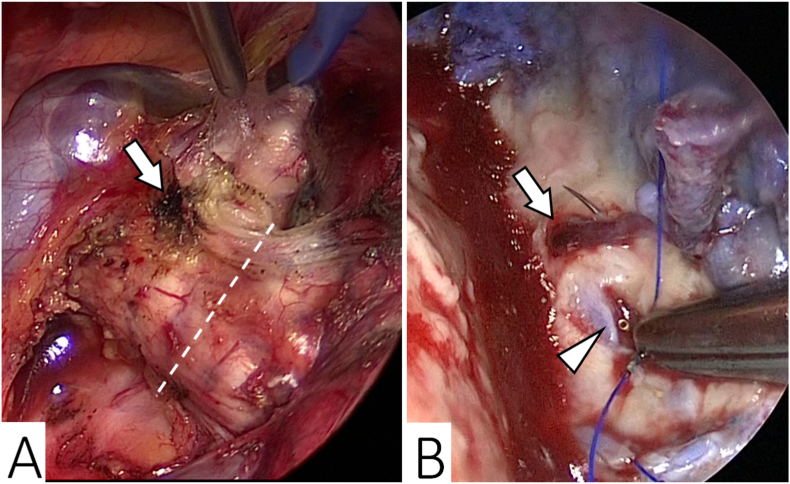
Findings from the retrospective video validations of each surgery. A: An electrical burn scar at the membranous portion of the intermedius bronchus (arrow) was visualized approximately 1 cm proximal to the bronchial stump (dotted line) during the right lower lobectomy. B: The suture needle penetrated the burned scar (arrow) through the preceding fistula (arrowhead) during the omentoplasty. Declarations of interest: none.

The present case suggested two clinical issues. One is that a metachronous BPF could develop following a preceding one. A retrospective validation of video recordings of the right lower lobectomy revealed an accidental electrical discharge by the electrocautery at the membranous portion of the intermedius bronchus (Fig. 2A). Furthermore, the suture thread penetrated the corresponding site during the omentoplasty (Fig. 2B). Those combined mechanical injuries to the bronchus might have resulted in the metachronous fistula. Further, positive pressure ventilation after the omentoplasty and malnutrition due to the low-fat diet with octreotide for the chylothorax treatment might have also adversely affected it. Octreotide reduces the blood flow and motility in the digestive system and inhibits the secretion of digestive enzymes, resulting in suppression of the absorption of fats, amino acids, and glucose [[14], [15], [16], [17]].

The other is that the harvested omental flap could have migrated even after being anchored in the thoracic cavity. Although omental migration in the abdominal cavity is well known [13,18,19], the present case suggested that the fixed omentum in the thoracic cavity had also migrated and yielded favorable results. The clinical outcome in the present case implies a novel therapeutic potential of a harvested omentum, which should be investigated by further experience accumulation in the future.

## 4 Conclusions

A metachronous BPF could develop due to both systemic and local mechanical factors. The harvested omental flap might migrate even after being anchored in the thoracic cavity.

## Decelerations

Provenance and peer review; Not commissioned, externally peer-reviewed.

## Sources of funding

We do not have any funding source, this manuscript is just a case report, not research.

## Ethical approval

As the manuscript was not a research study, we only have the patient consent for writing and other forms of publication.

## Consent

Written informed consent was obtained from the patient for publication of this case report and accompanying images. A copy of the written consent is available for review by the Editor-in-Chief of this journal on request.

## Registration of research studies

The manuscript is a case report and is not considered to be formal research involving participants.

## Conflict of interest statement

All authors do not have any conflicts of interests.

## Author contribution

SI and TN evaluated and interpreted the patient clinical data and were major contributors into the writing of the manuscript. AU and KF were contributors to interpreting the patient clinical data. All authors read and approved the final manuscript.

## References

[1] Asamura H., Naruke T., Tsuchiya R., Goya T., Kondo H., Suemasu K. (1992). Bronchopleural fistulas associated with lung cancer operations. Univariate and multivariate analysis of risk factors, management, and outcome. J. Thorac. Cardiovasc. Surg..

[2] Kitano M. (2008). Omentoplasty in thoracic surgery. General thoracic and cardiovascular surgery.

[3] Puskas J.D., Mathisen D.J., Grillo H.C., Wain J.C., Wright C.D., Moncure A.C. (1995). Treatment strategies for bronchopleural fistula. J. Thorac. Cardiovasc. Surg..

[4] Duan M., Chen G., Wang T., Zhang Y., Dong J., Li Z., Sui T. (1999). One-stage pedicled omentum majus transplantation into thoracic cavity for treatment of chronic persistent empyema with or without bronchopleural fistula. Eur. J. Cardio. Thorac. Surg. : official journal of the European Association for Cardio-thoracic Surgery.

[5] Shrager J.B., Wain J.C., Wright C.D., Donahue D.M., Vlahakes G.J., Moncure A.C., Grillo H.C., Mathisen D.J. (2003). Omentum is highly effective in the management of complex cardiothoracic surgical problems. J. Thorac. Cardiovasc. Surg..

[6] Agha R.A., Franchi T., Sohrabi C., Mathew G., Kerwan A. (2020). The SCARE 2020 guideline: updating consensus surgical CAse REport (SCARE) guidelines. Int. J. Surg..

[7] Okuda M., Go T., Yokomise H. (2017). Risk factor of bronchopleural fistula after general thoracic surgery: review article. General thoracic and cardiovascular surgery.

[8] Sirbu H., Busch T., Aleksic I., Schreiner W., Oster O., Dalichau H. (2001). Bronchopleural fistula in the surgery of non-small cell lung cancer: incidence, risk factors, and management. Ann. Thorac. Cardiovasc. Surg..

[9] Sonobe M., Nakagawa M., Ichinose M., Ikegami N., Nagasawa M., Shindo T. (2000). Analysis of risk factors in bronchopleural fistula after pulmonary resection for primary lung cancer. Eur. J. Cardio. Thorac. Surg..

[10] Clark J.M., Cooke D.T., Brown L.M. (2020). Management of complications after lung resection. Thorac. Surg. Clin..

[11] García-Gómez I., Goldsmith H.S., Angulo J., Prados A., López-Hervás P., Cuevas B., Dujovny M., Cuevas P. (2005). Angiogenic capacity of human omental stem cells. Neurol. Res..

[12] Goldsmith H.S., Griffith A.L., Kupferman A., Catsimpoolas N. (1984). Lipid angiogenic factor from omentum. JAMA.

[13] Meza-Perez S., Randall T.D. (2017). Immunological functions of the omentum. Trends Immunol..

[14] Lamberts S.W.J., Hofland L.J. (2019). Anniversary review: octreotide, 40 years later. Eur. J. Endocrinol..

[15] Kalomenidis I. (2006). Octreotide and chylothorax. Curr. Opin. Pulm. Med..

[16] Cheung Y., Leung M.P., Yip M. (2001). Octreotide for treatment of postoperative chylothorax. J. Pediatr..

[17] Markham K.M., Glover J.L., Welsh R.J., Lucas R.J., Bendick P.J. (2000). Octreotide in the treatment of thoracic duct injuries. Am. Surg..

[18] Morison R. (1906). Remarks on some functions of the omentum. Br. Med. J..

[19] Liu Y., Hu J.N., Luo N., Zhao J., Liu S.C., Ma T., Yao Y.M. (2021). The essential involvement of the omentum in the peritoneal defensive mechanisms during intra-abdominal sepsis. Front. Immunol..

